# Time-dependent ARMA modeling of genomic sequences

**DOI:** 10.1186/1471-2105-9-S9-S14

**Published:** 2008-08-12

**Authors:** Jerzy S Zielinski, Nidhal Bouaynaya, Dan Schonfeld, William O'Neill

**Affiliations:** 1 Department of Systems Engineering, University of Arkansas at Little Rock, Little Rock, AR, USA; 2 Department of Electrical and Computer Engineering, University of Illinois at Chicago, Chicago, IL, USA; 3 Department of Bioengineering, University of Illinois at Chicago, Chicago, IL, USA

## Abstract

**Background:**

Over the past decade, many investigators have used sophisticated time series tools for the analysis of genomic sequences. Specifically, the correlation of the nucleotide chain has been studied by examining the properties of the power spectrum. The main limitation of the power spectrum is that it is restricted to stationary time series. However, it has been observed over the past decade that genomic sequences exhibit non-stationary statistical behavior. Standard statistical tests have been used to verify that the genomic sequences are indeed not stationary. More recent analysis of genomic data has relied on time-varying power spectral methods to capture the statistical characteristics of genomic sequences. Techniques such as the evolutionary spectrum and evolutionary periodogram have been successful in extracting the time-varying correlation structure. The main difficulty in using time-varying spectral methods is that they are extremely unstable. Large deviations in the correlation structure results from very minor perturbations in the genomic data and experimental procedure. A fundamental new approach is needed in order to provide a stable platform for the non-stationary statistical analysis of genomic sequences.

**Results:**

In this paper, we propose to model non-stationary genomic sequences by a time-dependent autoregressive moving average (TD-ARMA) process. The model is based on a classical ARMA process whose coefficients are allowed to vary with time. A series expansion of the time-varying coefficients is used to form a generalized Yule-Walker-type system of equations. A recursive least-squares algorithm is subsequently used to estimate the time-dependent coefficients of the model. The non-stationary parameters estimated are used as a basis for statistical inference and biophysical interpretation of genomic data. In particular, we rely on the TD-ARMA model of genomic sequences to investigate the statistical properties and differentiate between coding and non-coding regions in the nucleotide chain. Specifically, we define a quantitative measure of randomness to assess how far a process deviates from white noise. Our simulation results on various gene sequences show that both the coding and non-coding regions are non-random. However, coding sequences are "whiter" than non-coding sequences as attested by a higher index of randomness.

**Conclusion:**

We demonstrate that the proposed TD-ARMA model can be used to provide a stable time series tool for the analysis of non-stationary genomic sequences. The estimated time-varying coefficients are used to define an index of randomness, in order to assess the statistical correlations in coding and non-coding DNA sequences. It turns out that the statistical differences between coding and non-coding sequences are more subtle than previously thought using stationary analysis tools: Both coding and non-coding sequences exhibit statistical correlations, with the coding regions being "whiter" than the non-coding regions. These results corroborate the evolutionary periodogram analysis of genomic sequences and revoke the stationary analysis' conclusion that coding DNA behaves like random sequences.

## Background

Understanding the statistical properties of genomic sequences helps recreate the dynamical processes that led to the current DNA structure, and determine gene-related diseases like cancer and Alzheimer disease. For instance, based on the view that non-coding DNA exhibits long-range correlations [1-6], Li [7] proposed an expansion-modification model of gene evolution. The model incorporates the two basic features of DNA evolution: (i) sequence elongation due to gene duplication and (ii) mutations. It can be shown that the limiting sequence created by this dynamical process exhibits a long-range correlation structure, as attested by a 1/*f*^*α *^spectrum, where the exponent *α *is a function of the probability of mutation. To understand the relationship between the DNA correlation structure and possible gene abberations, Dodin et al. [8] designed a simple correlation function intended to visualize the regular patterns encountered in DNA sequences. This function is used to revisit the intriguing question of triplet repeats with the aim of providing a visual estimate of the propensity of genes to be highly expressed and/or to lead to possible aberrant structures formed upon strand slippage.

Statistical analysis of genomic sequences has, however, relied, for a long time, on signal processing techniques for stationary signals (correlation and power spectrum) [2,4,9,10], and statistical tools for slowly-varying trends within stationary signals (Detrended Fluctuation Analysis or DFA) [1,5,6]. Stationarity can be argued as a valid assumption for time-series of short duration. However, such an assumption rapidly loses its credibility in the enormous databases maintained by various genome projects. Standard statistical tests (e.g., Priestley's test for stationarity) have been used to verify that genomic sequences are not stationary and the nature of their non-stationarity varies and is often more complex than a simple trend [11,12]. Subsequently, more recent analysis of genomic data [1] has relied on time-varying power spectral methods (the evolutionary spectrum and periodogram) to capture the statistical characteristics of genomic sequences [11,12]. The main difficulty in using time-varying spectral methods is that they are extremely unstable and very noisy. Typically, the power spectrum and the evolutionary spectrum are averaged over time in order to obtain smooth and less jittery curves. Moreover, as was pointed out in [13], the evolutionary spectrum is restricted to the class of oscillatory processes. A stochastic process, *X*(*t*), is oscillatory if it has a representation of the form

(1) *X*(*t*) = ∫ *A*(*t*, *λ*)*e*^2*iπλt *^*dZ*(*λ*),

Where *Z*(*λ*) is an orthogonal increment process, and the evolutionary power spectrum of the process is defined by *P *(*t*, *λ*) = |*A*(*t*, *λ*)|^2^. This definition of the evolutionary power spectrum has the following disadvantages [13]:

**(i) **It is not uniquely defined for a given non-stationary process.

**(ii) **The estimation procedure for the evolutionary spectrum depends greatly on the nature of theamplitude function *A*(*t*, *λ*), which is not known a priori.

**(iii) **An increase in the number of observations does not provide added information on the local behavior of the evolutionary spectrum, and thus does not improve estimation accuracy.

We propose to model non-stationary genomic sequences by a time-dependent autoregressive moving average (TD-ARMA) process. Cramer [14] showed that a non-stationary process still possesses a Wold decomposition in terms of its innovation and its generating system. However, the linear system generating the non-stationary signal, *x*(*t*), when driven by the innovation, *w*(*t*), is no longer shift-invariant; the parameters of the impulse response, *h*_*u*_, of this system are time-dependent so that

(2) $$x(t)=\sum_{u=0}^{\infty }h_{u}(t)w(t-u).$$

The existence of a time-varying ARMA representation of this process is ensured by two theorems due, independently, to Grenier [15] and Huang and Aggarwal [16]. The uniqueness of the TD-ARMA representation is obtained by constraining the ARMA model to be invertible, but this leads to conditions on the time-varying impulse response {*h*_*u*_(*t*)} and its inverse (namely to be absolutely summable at any time *t*), which cannot be easily expressed in terms of the time-dependent coefficients of the ARMA model. In this paper, we estimate the time-dependent coefficients of the general TD-ARMA model using mean-squares, least-squares and recursive least-squares algorithms. The mean-squares estimation leads to generalized Yule-Walker type equations [15]. Once the non-stationary parameters are estimated (as time series), we use them to provide a basis for statistical inference by defining an index of randomness, which quantitatively assesses how close the non-stationary signal is to white noise. Our simulation results on various gene sequences show that (i) both the coding and non-coding segments of a gene are not random, and (ii) the coding segments are "closer" to random sequences than non-coding segments. Our results support the view that both coding and non-coding sequences are not random [11,12,9,17-20], and revokes the stationary study that maintains that non-coding DNA sustains long-range correlations whereas coding DNA behaves like random sequences [1-3,5,6,10].

## Methods

### Numerical representation of genomic sequences

**Figure 1:**
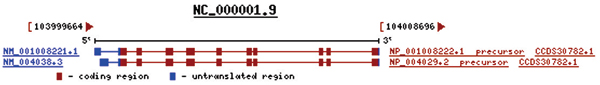
**Gene Structure**. Gene structure of the Human gene 276 located in chromosome 1: The boxes correspond to the exons (coding regions), and the lines between them represent the introns (non-coding regions). The total length of the gene is *N *= 8208 bases, including 1536 coding bases and 6672 non-coding bases.

**Figure 2:**
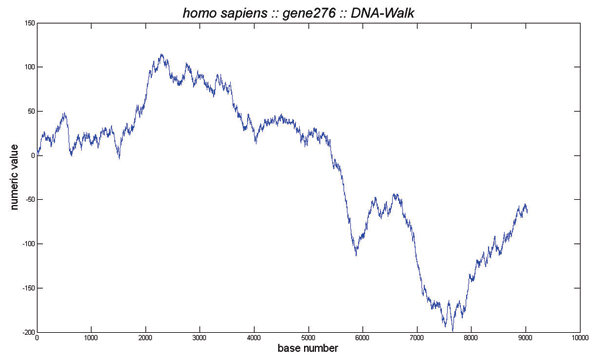
**DNA Walk**. DNA walk of the Human gene 276.

Converting the DNA sequence into a digital signal offers the opportunity to apply powerful signal processing methods for the handling and analysis of genomic information. This is, however, not an easy task as the analysis results might depend on the chosen map. Various numerical mappings have been adopted in the literature. To cite few, Peng et al. [1] construct a one-dimensional map of nucleotide sequences onto a walk, *u*(*i*), which they termed "DNA walk". The DNA walk is defined by the rule that the walker steps up (*u*(*i*) = +1) if a pyrimidine resides at position *i*, and steps down (*u*(*i*) = -1) otherwise. Voss [9] represents a DNA sequence by four binary indicator sequences, which indicate the locations of the four nucleotides A, T, C and G. Berthelsen et al. [21] proposed a two-dimensional representation of DNA sequences, characterized by a Hausdorff dimension (also called fractal dimension) that is invariant under (i) complementarity, (ii) reflection symmetry, (iii) compatibility and (iv) substitution symmetry of A͘T and C↔G. The corresponding embedding assignment is given by A = (-1; 0), T = (1; 0), C = (0; -1) and G = (0; 1). In this paper, since we are interested in time-dependent ARMA modeling of one-dimensional non-stationary genomic sequences, we adopt the widely used "DNA walk" map proposed by Peng et al [1]. The DNA walk provides a nice graphical representation for each gene. For instance, Figure 1 shows the structure of the Human gene 276 located in chromosome 1, and its DNA walk is displayed in Fig. 2.

### Time-dependent ARMA model

Grenier [22] showed that a discrete non-stationary signal, {*x *[*n*]}, can be represented by finite-order time-varying ARMA processes of the form

(3) $$\begin{array}{cc} x[n]+\sum_{i=1}^{p}a_{i}[n-i]\;x[n-i]=w[n]+\sum_{i=1}^{q}b_{i}[n-i]\;w[n-i], & n=0,\cdots ,N-1, \end{array}$$

where *N *is the length of the sequence *x *[*n*], *a*_*i *_[*n*] and *b*_*i *_[*n*] are the time-dependent model parameters, *p *and *q *are the model orders and *w *[*n*] is a white noise process. Observe that the coefficients *a*_*i *_[*n*] and *b*_*i *_[*n*] appear with an argument *n *- *i *depending on *i*. This is purely arbitrary since any time origin can be chosen, without restraining the generality of the model. We assume that the time-dependent coefficients *a*_*i *_[*n*] and *b*_*i *_[*n*] can be expressed as linear combinations of some basis functions ${\left\{f_{k}[n]\right\}}_{k=0}^{m}$,

(4) $$a_{i}[n]=\sum_{k=0}^{m}c_{i,k}f_{k}[n]$$

(5) $$b_{i}[n]=\sum_{k=0}^{m}d_{i,k}f_{k}[n]$$

The advantage of the basis parametrization is clear from the fact that the identification of the time-dependent coefficients *a*_*i *_[*n*] and *b*_*i *_[*n*] reduces to the identification of the constant coefficients ${\left\{c_{i,k}\right\}}_{k=0}^{m}$ and ${\left\{d_{i,k}\right\}}_{k=0}^{m}$, and therefore the linear non-stationary problem reduces to a linear time-invariant problem. The basis functions ${\left\{f_{k}[n]\right\}}_{k=0}^{m}$ do not have to be limited to the standard choices of Legendre, Fourier, or the prolate spheroidal basis but can also take advantage of any prior information, such as the presence of a jump in the coefficients at a known instant [22].

### Estimation of the time-dependent ARMA coefficients

From Eqs. (4) and (5), the unknown parameters of the TD-ARMA model are the weights of the linear combinations defining each time-varying parameter. The linearity is the key to the algorithms proposed in this paper. We will derive mean-squares, least-squares and recursive least-squares solutions to estimate the time-dependent coefficients ${\left\{a_{i}[n]\right\}}_{i=1}^{p}$ and ${\left\{b_{j}[n]\right\}}_{j=1}^{q}$.

#### Mean-squares estimation

Define the process

(6) $$\begin{array}{cc} v[n]=x[n]+\sum_{i=1}^{p}a_{i}[n-i]\;x[n-i]=w[n]+\sum_{i=1}^{q}b_{i}[n-i]\;w[n-i], & n=0,\cdots N-1, \end{array}$$

and define the vector

(7) *X *[*n*] = [*f*_0_[*n*]*x*[*n*], ⋯, *f*_*m *_[*n*]*x*[*n*]]^*t*^,

where the symbol ^*t *^stands for the transpose of a vector or a matrix. It is possible to derive for this process orthogonality conditions similar to the stationary ARMA model conditions [23]. Observe that the process *v *[*n*], defined in Eq. (6), is orthogonal to [*w*[*n *- *q *- 1], *w *[*n *- *q *- 2], ⋯]; hence, it is orthogonal to *x *[*n *- *q *- *i*] for all *i *> 0, and orthogonal to *X *[*n *- *q *- *i*] for all *i *> 0 [22]. This orthogonality condition leads to a generalized Yule-Walker equation [22]

(8) $$E\left(\left[\begin{array}{c} X[n-q-1]\\ \vdots \\ X[n-q-p] \end{array}\right]{\left[X{\left[n-1\right]}^{t}\cdots X{\left[n-p\right]}^{t}\right]}^{t}\right)\theta =-E\left(\left[\begin{array}{c} X[n-q-1]\\ \vdots \\ X[n-q-p] \end{array}\right]\cdot x[n]\right)$$

Although the process *x *[*n*] is non-stationary, the stationarity and ergodicity of the process *w *[*n*], together with the linearity of the model, allow us to replace in Eq. (8) the expectation by a summation. However, although consistent, the above estimator is often considered a poor one [22].

#### Least-squares estimation

Equations (4) and (5) can be written in vector format as follows

*a*_*i *_[*n*] = **f**^**t **^[*n*] **c**_**i**_,   and   *b*_*i *_[*n*] = **f**^**t **^[*n*] **d**_**i**_,

where

$$\begin{array}{ccc} f[n]=\left[\begin{array}{c} f_{0}[n]\\ \vdots \\ f_{m}[n] \end{array}\right], & c_{i}=\left[\begin{array}{c} c_{i,0}\\ \vdots \\ c_{i,m} \end{array}\right], & d_{i}=\left[\begin{array}{c} d_{i,0}\\ \vdots \\ d_{i,m} \end{array}\right]. \end{array}$$

Define

**u**^**t **^[*n*] = *x *[*n*] **f**^**t **^[*n*],   and   **v**^**t **^[*n*] = *w *[*n*] **f**^**t **^[*n*].

Then, we have

$$\begin{array}{c} a_{i}[n-i]\;x[n-i]=u^{t}[n-i]\;c_{i}\\ b_{i}[n-i]\;w[n-i]=v^{t}[n-i]\;d_{i} \end{array}$$

Using this vector notation, Eq. (3) can be written as

(9) *x *[*n*] + **u**^*t *^[*n *- 1] **c**_**1 **_+ ⋯ + **u**^*t *^[*n *- *p*] **c**_**p **_= *w *[*n*] + **v**^*t *^[*n *- 1] **d**_**1 **_+ ⋯ + **v**^*t *^[*n *- *q*] **d**_**q**_

Or equivalently

(10) *x *[*n*] + *φ*^*t *^[*n*] *θ *= *w *[*n*],

where *φ*^*t *^[*n*] is the row vector

*φ*^*t *^[*n*] = [**u**^*t *^[*n *- 1], ⋯, **u**^*t *^[*n *-*p*], - **v**^*t *^[*n *- 1], ⋯, **v**^*t *^[*n *-*q*]],

and

***θ ***= [**c**_**1**_, ⋯,**c**_**p**_, **d**_**1**_, ⋯, **d**_**q**_]^*t*^.

Observe that the vector *θ *contains all the unknowns of the TD-ARMA model. Writing Eq. (10) for *n *= 0, 1, ⋯, *N *- 1 leads to

(11) **x = Φ *θ *+ w**,

where

$$\begin{array}{ccc} \Phi =\left[\begin{array}{c} -\varphi^{t}[0]\\ \vdots \\ -\varphi^{t}[N-1] \end{array}\right], & x=\left[\begin{array}{c} x[0]\\ \vdots \\ x[N-1] \end{array}\right], & w=\left[\begin{array}{c} w[0]\\ \vdots \\ w[N-1] \end{array}\right]. \end{array}$$

The least-squares solution of Eq. (11) is given by

(12) ***θ *= (Φ^*t *^Φ)^-1 ^Φ^*t *^x**

To overcome the computational complexity associated with the least-squares estimation (involving in particular the inversion of a square (*m *+ 1)(*p *+ *q*) × (*m *+ 1)(*p *+ *q*) matrix), we opted for a recursive least-squares estimation as follows.

#### Recursive least-squares estimation

The recursive least squares algorithm is summarized as [24]

(13) $$\hat{\theta }[n]=\hat{\theta }[n-1]+L[n]\;\{x[n]+\varphi^{t}[n]\hat{\theta }[n-1]\}$$

(14) $$L[n]=-\frac{P[n-1]\;\varphi [n]}{1+\varphi^{t}[n]\;P[n-1]\;\varphi [n]}$$

(15) $$P[n]=P[n-1]-\frac{P[n-1]\;\varphi [n]\;\varphi^{t}[n]\;P[n-1]}{1+\varphi^{t}[n]\;P[n-1]\;\varphi [n]}$$

The initial conditions can be chosen arbitrarily.

### Index of randomness

Over the past decade, there has been a flow of conflicting papers about the long-range power-law correlations detected in eukaryotic DNA [1-3,5,6,9-12,17-20]. The controversy is generated by conflicting views that either advocate that non-coding DNA sustains long-range correlations whereas coding DNA behaves like random sequences [1,10,3,5,6], or maintains that both coding and non-coding DNA exhibit long-range power-law correlations [11,12,9,17-20]. Based on the analysis of the time-dependent power spectrum of genomic sequences, Bouaynaya and Schonfeld [11,12] showed that the statistical differences between coding and non-coding sequences are more subtle than previously concluded using stationary analysis tools. In fact they found that both coding and non-coding sequences are non-random. However, coding sequences are "whiter" than non-coding sequences.

We propose to qualitatively assess the degree of randomness of both coding and non-coding sequences using the time-dependent ARMA coefficients *a*_*i *_[*n*] and *b*_*i *_[*n*]. Consider the system function, *H *(*z*), of a stationary ARMA model (whose coefficients *a*_*i *_and *b*_*i *_are constant, i.e., independent of time). We know that

(16) $$H(z)=\frac{\sum_{k=0}^{q}b_{k}z^{-k}}{\sum_{k=0}^{p}a_{k}z^{-k}}=\frac{\prod_{k=1}^{q}\left(1-r_{k}z^{-1}\right)}{\prod_{k=1}^{p}\left(1-p_{k}z^{-1}\right)},$$

where ${\left\{r_{k}\right\}}_{k=1}^{q}$ (resp. ${\left\{p_{k}\right\}}_{k=1}^{p}$) are the zeros (resp. poles) of the system function. From the fact that a stationary white noise process has a at spectrum, we observe that the closer (in L_2 _distance) the zeros and poles are, the flatter is the spectrum of the process. Following the same reasoning locally for non-stationary processes, we define the curve of randomness, *CR *[*n*], of the non-stationary process *x *[*n*] by

(17) $$\{\begin{array}{ll} CR[n]=\min_{\left(r_{k}[n],p_{k}[n]\right)}\left(\frac{1}{q}\sum_{k=1}^{q}\left|r_{k}[n]-p_{k}[n]\right|+\frac{1}{p-q}\sum_{k=q+1}^{p}\left|p_{k}[n]\right|\right), & \text{if }p>q;\\ CR[n]=\min_{\left(r_{k}[n],p_{k}[n]\right)}\left(\frac{1}{p}\sum_{k=1}^{p}\left|r_{k}[n]-p_{k}[n]\right|+\frac{1}{p-q}\sum_{k=p+1}^{q}\left|r_{k}[n]\right|\right), & \text{if }q>p;\\ CR[n]=\min_{\left(r_{k}[n],p_{k}[n]\right)}\left(\frac{1}{p}\sum_{k=1}^{p}\left|r_{k}[n]-p_{k}[n]\right|\right), & \text{if }p=q. \end{array}$$

where the minimum is taken over all pairs (*r*_*k *_[*n*], *p*_*k *_[*n*]). Observe that the case *p *<*q *is obtained from the *p *> *q *case by interchanging the roles of *r*_*k *_and *p*_*k*_, and the indices *p *and *q*. The curve of randomness defined in Eq. (17) is a measure of how close the zeros and the poles of the system function are, and therefore, is a measure of how flat the system function is, and how close is the process from a white noise. The index of randomness, *IR*(*p*, *q*), of a TD-ARMA(*p*, *q*), is then defined as the average of the curve of randomness, i.e.,

(18) $$IR(p,q)=\frac{1}{N}\sum_{n=0}^{N-1}CR[n].$$

In particular, the index of randomness of a TD-ARMA(1,1) (*x *[*n*] + *a*[*n *- 1]*x*[*n *- 1] = *w*[*n*] + *b*[*n*]*w*[*n *- 1]) is given by

(19) $$IR(1,1)=\frac{1}{N}\sum_{n=0}^{N-1}\left|a[n]-b[n]\right|.$$

Observe that the index of randomness of a white noise process is equal to zero. We say that the sequence *x*_1 _[*n*] with index of randomness *IR*_1 _is more random than the sequence *x*_2 _[*n*] with index of randomness *IR*_2 _if the index of randomness of the former is lower than the index of randomness of the latter, i.e., *IR*_1 _<*IR*_2_.

## Results

**Figure 3:**
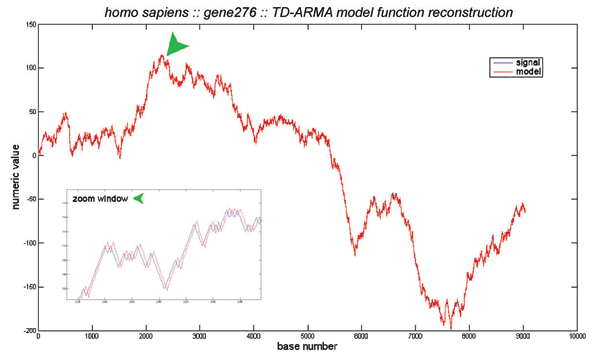
**TD-ARMA modeling**. TD-ARMA modeling of the Human gene 276: The blue signal is the DNA walk, and the red signal is the TD-ARMA(1,1) fitted signal. The TD-ARMA(1,1) model accurately fits the genomic signal.

**Figure 4:**
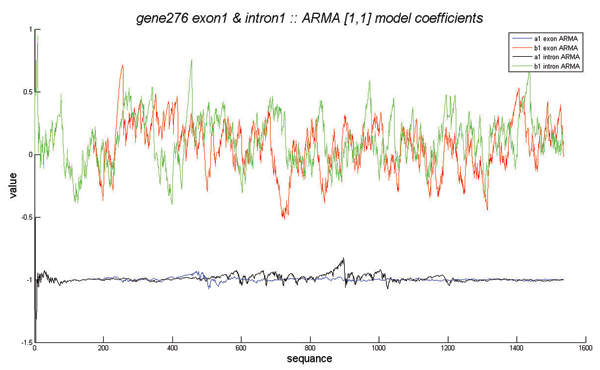
**TD-ARMA coefficients estimation**. Estimation of the TD-ARMA(1,1) coefficients of the Human gene 276. The TD-ARMA(1,1) model is given by *x *[*n*] + *a *[*n *- 1] *x *[*n *- 1] = *w *[*n*] + *b *[*n *- 1] *w *[*n *- 1]. The blue and black (resp. red and green) curves depict the time series *a*[*n*] (resp. *b*[*n*]) for the coding and non-coding regions of the gene, respectively.

**Figure 5:**
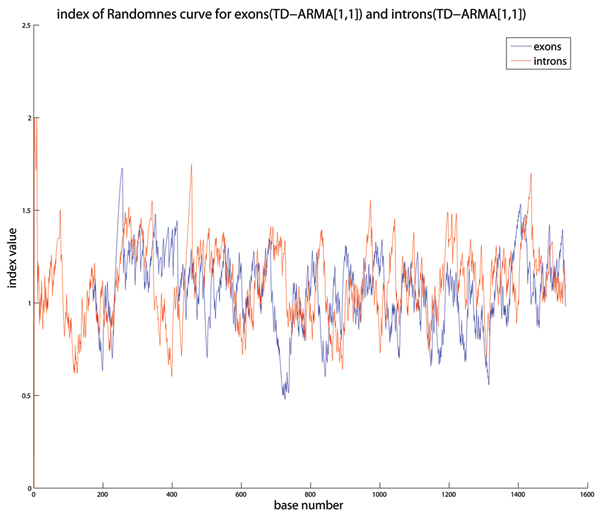
**Curve of randomness**. The curves of randomness of the coding and non-coding regions of the Human gene 276 are shown in blue and red, respectively. The index of randomness of the coding sequence is equal to 1.0603, whereas its corresponding value for the non-coding sequence is equal to 1.0627.

All genome sequences considered in this paper have been extracted from the NIH website . The algorithms were implemented in MATLAB. The DNA sequences were mapped to numerical sequences using the purine-pyrimidine DNA walk proposed in [1]. In our simulations, the recursive least squares algorithm was found to best estimate the time-dependent coefficients of the TD-ARMA model. We used the MATLAB function *rarmax*, which implements the recursive least-squares algorithm for TD-ARMA models. The choice of the orders *p *and *q *of the model were determined experimentally as follows: For each genomic sequence, we computed 100 TD-ARMA models corresponding to the orders (1, 1) up to (10, 10). The best model was chosen to be the one that minimizes the average squared error between the actual and the fitted sequences. Our extensive simulations on various DNA sequences from different organisms show that most of the sequences are best fitted with low-order TD-ARMA models, e.g., TD-ARMA(1,1), TD-ARMA(1,2) and TD-ARMA(2,1). Figure 3 shows the DNA walk of the Human gene 276 and its TD-ARMA(1,1) fitted sequence. Observe that the TD-ARMA(1,1) model accurately fits this gene sequence. The estimated time-varying coefficients *a *[*n*] and *b *[*n*] are displayed in Fig. 4 for both the coding and non-coding regions of this gene. Their statistical differences are not clear from the plot of the time-series coefficients. The curves of randomness of the coding and non-coding regions are displayed in Fig. 5. Table 1 shows the index of randomness of various gene sequences. For concise representation, the column titles have been abbreviated as follows: "C. length" (resp."N.C. length") denotes the length (in base pairs) of the coding (resp. non-coding) segment of the gene. The total length of the gene is the sum of the lengths of its coding and non-coding regions. "C. (*p*, *q*)" (resp. "N.C. (*p*, *q*)") denotes the optimal TD-ARMA parameters (*p*, *q*) for the coding (resp. non-coding) region of the gene. Finally, "C. IR" (resp. "N.C. IR") is the index of randomness of the coding (resp. non-coding) segment of the gene. Observe that, in all considered genes, the index of randomness of both the coding and non-coding segments are strictly positive, and the index of randomness of the coding region is consistently lower than the index of randomness of the non-coding region (recall that the index of randomness of a white noise is zero). These observations bring to bear two important conclusion: (i) Both the coding and non-coding sequences are not random, as attested by an index of randomness greater than zero. (ii) The coding sequences are "whiter" than the non-coding sequences. This conclusion revokes previous work on statistical correlation of DNA sequences, which, based on stationary time-series analysis, presumed that coding DNA behaves like random sequences [1-3,5,6,10]; and supports the conflicting view that both coding and non-coding sequences are not random [11,12,9,17-20]. In particular, our conclusion is in accordance with the evolutionary periodogram analysis conducted in [11,12].

**Table 1:** Index of Randomness of the Coding and Non-Coding segments of Various Gene Sequences

Gene NIH accession number	C. length	C. (*p*, *q*)	C. IR	N.C. length	N.C. (*p*, *q*)	N.C. IR
Ashbya gossypii (fungus) AE016815	180953	(1,1)	0.9466	674919	(1,1)	0.9860
Aspergillus fumigatus (form of fungus) CM000169	1227993	(2,1)	0.9870	1835394	(1,1)	1.0683
Candida albicans (form of yeast) AP006852	373390	(1,1)	1.0282	570789	(1,1)	1.0429
Candida albicans AP006852	373390	(1,1)	1.0282	570789	(3,1)	1.0429
fission yeast GI:157310483	753661	(1,1)	1.0402	1654671	(1,1)	1.0642
fruit fly AE002620	21399	(1,1)	1.0084	1222832	(1,2)	1.1075
fruit fly AE002725	11316	(1,1)	1.0145	659655	(1,1)	1.0320
Homo sapiens hs-gene277 NG-004750	1639	(1,1)	1.0688	6573	(1,1)	1.0808
Homo sapiens hs-gene276 NG-004750	1536	(1,1)	1.0603	6672	(1,1)	1.0627

## Conclusion

In this paper, we modelled the non-stationary genomic sequences by a time-dependent autoregressive moving average (TD-ARMA) model. By expressing the time-dependent coefficients as linear combinations of parametric basis functions, we were able to transform a linear non-stationary problem into a linear time-invariant problem. Subsequently, we proposed three methods to estimate the time-dependent coefficients: Mean -square, least-squares, and recursive least-squares algorithms. Based on the estimated TD-ARMA coefficients, we defined an index of randomness to quantify the degree of randomness of both coding and non-coding sequences. We found that both coding and non-coding sequences are not random. However, a higher index of randomness attests that coding sequences are "whiter" than non-coding sequences. These results corroborate the evolutionary periodogram analysis of genomic sequences performed in [11] and [12], and revoke the stationary analysis' conclusion that coding DNA behaves like random sequences.

## Competing interests

The authors declare that they have no competing interests.

## Authors' contributions

JSZ derived the different estimation algorithms of the TD-ARMA parameters and performed the simulations. NB proposed the use of the non-stationary analysis and the index of randomness as a basis for statistical inference and biophysical interpretation of genomic data, derived the different estimation algorithms of the TD-ARMA parameters, and drafted the manuscript. DS proposed the use of the non-stationary analysis and the index of randomness as a basis for statistical inference and biophysical interpretation of genomic data and derived the different estimation algorithms of the TD-ARMA parameters. WO proposed the use of TD-ARMA modeling as a non-stationary model of genomic sequences. All authors read and approved the final manuscript.
